# Gut Microbiota in Children Hospitalized with Oedematous and Non-Oedematous Severe Acute Malnutrition in Uganda

**DOI:** 10.1371/journal.pntd.0004369

**Published:** 2016-01-15

**Authors:** Kia Hee Schultz Kristensen, Maria Wiese, Maren Johanne Heilskov Rytter, Mustafa Özçam, Lars Hestbjerg Hansen, Hanifa Namusoke, Henrik Friis, Dennis Sandris Nielsen

**Affiliations:** 1 Department of Nutrition, Exercise and Sports, Section of Child Nutrition and International Nutrition, Faculty of Science, University of Copenhagen, Copenhagen, Denmark; 2 Department of Food Science, Section of Food Microbiology, Faculty of Science, University of Copenhagen, Copenhagen, Denmark; 3 Department of Pediatrics, Copenhagen University Hospital, Rigshospitalet, Copenhagen, Denmark; 4 Department of Environmental Science, Section of Environmental Microbiology and Biotechnology, University of Aarhus, Roskilde, Denmark; 5 Department of Pediatrics and Child Health, Mwanamugimu Nutrition Unit, Mulago Hospital, Kampala, Uganda; University of California, San Diego School of Medicine, UNITED STATES

## Abstract

**Background:**

Severe acute malnutrition (SAM) among children remains a major health problem in many developing countries. SAM manifests in both an oedematous and a non-oedematous form, with oedematous malnutrition in its most severe form also known as kwashiorkor. The pathogenesis of both types of malnutrition in children remains largely unknown, but gut microbiota (GM) dysbiosis has recently been linked to oedematous malnutrition. In the present study we aimed to assess whether GM composition differed between Ugandan children suffering from either oedematous or non-oedematous malnutrition.

**Methodology/Principal Findings:**

As part of an observational study among children hospitalized with SAM aged 6–24 months in Uganda, fecal samples were collected at admission. Total genomic DNA was extracted from fecal samples, and PCR amplification was performed followed by Denaturing Gradient Gel Electrophoresis (DGGE) and tag-encoded 16S rRNA gene-targeted high throughput amplicon sequencing. Alpha and beta diversity measures were determined along with ANOVA mean relative abundance and G-test of independence followed by comparisons between groups. Of the 87 SAM children included, 62% suffered from oedematous malnutrition, 66% were boys and the mean age was 16.1 months. GM composition was found to differ between the two groups of children as determined by DGGE (p = 0.0317) and by high-throughput sequencing, with non-oedematous children having lower GM alpha diversity (p = 0.036). However, beta diversity analysis did not reveal larger differences between the GM of children with oedematous and non-oedematous SAM (ANOSIM analysis, weighted UniFrac, R = -0.0085, p = 0.584; unweighted UniFrac, R = 0.0719, p = 0.011).

**Conclusions/Significance:**

Our results indicate that non-oedematous SAM children have lower GM diversity compared to oedematous SAM children, however no clear compositional differences were identified.

## Author Summary

Severe acute malnutrition (SAM) is a major health problem, responsible for many deaths among young children in low-income countries. SAM manifests as oedematous or non-oedematous malnutrition. Oedematous malnutrition, also known as kwashiorkor, is a life-threatening condition, and even today we do not understand why some children develop oedema with malnutrition. Recently, an association between gut microbiota dysbiosis and oedematous malnutrition has been suggested. However, it remains unknown whether the gut microbiota differs between children with oedematous and non-oedematous malnutrition. In the present study, we collected fecal samples from children with SAM with and without oedema and analyzed the gut microbiota composition. We found that the pattern of bacteria was different in the two types of malnutrition, and that fewer different types of bacteria, on average, were present in the guts of non-oedematous children. However, we could not identify any specific type of bacteria that explained this difference. These results may contribute to the understanding of oedematous SAM, and inspire to further research into better ways of treatment of these very ill children.

## Introduction

Malnutrition remains a major problem in developing countries with moderate and severe acute malnutrition (SAM) accounting for 12.6% of total deaths of children younger than 5 years of age [1]. SAM manifests itself as two clinical phenotypes, namely oedematous and non-oedematous SAM. The factors determining the clinical phenotype remain unresolved. Oedematous malnutrition is a life-threatening condition and is, in its most severe form, kwashiorkor, characterized by generalized bilateral oedema, enlarged steatotic liver, skin changes and apathy [2]. Although kwashiorkor has been known since the 1930s [2], previous hypotheses about protein deficiency and oxidative stress do not explain the condition [3–6].

Recent studies suggest a link between gut microbiota (GM) and malnutrition [7], with several studies reporting predominance of pathogenic intestinal bacteria in the guts of malnourished children compared to healthy controls [8–11]. Concordantly, it has been suggested that pathogenic overload leads to persistent enteric inflammation, increased permeability and nutrient malabsorption [12]. A direct relationship between malnutrition and GM was demonstrated by transplanting fecal samples from Malawian twin pairs discordant for oedematous malnutrition, kwashiorkor, into germ free mice [7], but while it seems to be well-established that GM dysbiosis is associated with malnutrition, it remains unknown whether GM differs between the two types of malnutrition, oedematous and non-oedematous SAM, respectively.

In the present study, we hypothesized that GM composition differs between the two clinical types of SAM, suggesting a possible correlation between GM and the development of the two phenotypes.

## Materials and Methods

### Study design and population

The study was conducted in a subsample of children included in the observational study FeedSAM. The main study included 120 children aged 6–59 months admitted for treatment of SAM, of which we included 87 children aged 6–24 months in the substudy. Inclusion took place from October 2012 to March 2013. All children were recruited at Mwanamugimu Nutrition Unit (MNU), Mulago Hospital, Kampala, Uganda. The children received standard treatment of SAM according to the Ugandan national guidelines Integrated Management of Acute Malnutrition (IMAM), based on the World Health Organization (WHO) protocol [13]. The children were given therapeutic diets F75 and F100 (Nutriset, France) along with empiric antibiotics (ampicillin and gentamicin). In case of dehydration, the children received oral rehydration solution for malnutrition (ReSoMal, Nutriset, France). When the children were clinically well, had regained good appetite and lost all oedema, they were discharged for outpatient treatment with ready-to-use therapeutic food (RUTF, Nutriset, France). HIV testing was offered to all mothers and children, and done as recommended by WHO [14].

Inclusion criteria were age 6–24 months, presence of SAM defined by weight-for-height (WHZ) < -3 standard deviations and/or mid-upper-arm-circumference (MUAC) < 11.5 cm and/or bilateral pitting oedema [15]. Other criteria were residing close enough for follow-up and having a caretaker that provided informed consent to participate. Exclusion criteria were shock, severe respiratory insufficiency, severe bleeding, very severe anemia equivalent to hemoglobin level < 4 g/dl, weight < 4.5 kg, previous admission due to SAM in the last 6 months, congenital syndromes and malignancies.

### Ethical considerations

The study was approved by the Ethical Review Board of the School of Public Health, Makerere University, by the Uganda National Council of Science and Technology (UNCST), and a consultative approval was given by the Danish National Committee on Biomedical Research Ethics. Parents or guardians of all participating children gave informed consent, and signed an informed consent form. Information was given both orally and in writing, in English and Luganda.

### Collection of fecal samples

Fecal samples were collected as soon as possible at admission. For collection, small plastic bags and spoons were used. The samples were manually homogenized and immediately transferred to 2.0 mL cryo tubes and frozen at -20°C. For logistic reasons, samples collected in the evening or at night were stored at 5°C until morning and then frozen. At the end of every week, samples were transferred to -80°C, and after completion of the study shipped on dry ice to University of Copenhagen, Department of Food Science, Denmark, for analysis.

### DNA extraction

Total DNA was extracted from fecal samples using the QIAamp DNA Stool Mini Kit (Qiagen, Hilden, Germany) according to the manufacturer’s instructions [16] but with an initial bead-beating step using a FastPrep apparatus (QBiogene, MP Biomedicals, Ilkirch, France) to increase bacterial lysis. Quality and concentration of extracted DNA were confirmed using a NanoDrop 1000 Spectrophometer (Thermo Scientific, USA). The extracted DNA was stored at -20°C until later use.

### Denaturing Gradient Gel Electrophoresis

Denaturing Gradient Gel Electrophoresis (DGGE) was used as a screening tool for determining GM differences. Using the V3-region of the 16S rRNA gene as PCR target, DGGE was carried out as previously described using a denaturing gradient of 30–65% denaturant on an INGENYphorU-2 unit [17, 18]

### 16S rRNA gene tag-encoded amplicon sequencing

The V3 and V4 regions of the 16S rRNA gene were amplified using primers compatible with the Nextera Index Kit (Illumina, CA, USA) (NXt_V3-V4_F 5’-TCGTCGGCAGC GTCAGATGTGTATAAG AGACAGCCTAYGGGRB GCASCAG-3’ and NXt_V3-V4_R 5’-GTCTCGTGGGCTCGGAGATGTGTATAAGAGACAGGGACTACNNGGGTATCTAAT-3’; adapters in bold). PCR reactions containing 12 μl AccuPrimeTM SuperMix II (Life Technologies, CA, USA), 0.5 μl of each primer (10 μM), 5 μl of genomic DNA (~10 ng/μl), and nuclease-free water to a total volume of 20 μl were run on a SureCycler 8800 (Agilent, CA, USA). Cycling conditions applied were: Denaturation at 95°C for 2 min; 35 cycles of 95°C for 15 s, 55°C for 15s and 68°C for 40 s; followed by final elongation at 68°C for 5 min. To incorporate primers with adapters and indexes, PCR reactions contained 12 μl Phusion High-Fidelity PCR Master Mix (Thermo Fisher Scientific, USA, MA), 2 μl corresponding P5 and P7 primer (Nextera Index Kit), 2 μl PCR product and nuclease-free water for a total volume of 25 μl. Cycling conditions applied were: 98°C for 1 min; 12 cycles of 98°C for 10 s, 55°C for 20 s and 72°C for 20 s; elongation at 72°C for 5 min. The amplified fragments with adapters and tags were purified using AMPure XP beads (Beckman Coulter Genomic, CA, USA). Prior to library pooling clean constructs were quantified using a Qubit fluorometer (Invitrogen, Carlsbad, CA, USA) and then pooled in approximately equal concentrations to ensure even representation of reads per sample followed by 250 bp pair-ended MiSeq (Illumina) sequencing performed according to the instructions of the manufacturer.

The raw dataset containing pair-ended reads with corresponding quality scores was trimmed using CLC Genomic Workbench (CLC bio, Arhus, Denmark). Trimming settings were set to low quality limit of 0.01, with no ambiguous nucleotides allowed, and trimming off the primer sequences. Merging overlapped reads was performed using the “Merge overlapping pairs” tool using default settings. The Quantitative Insight Into Microbial Ecology (QIIME) tool (version. 1.8.0; Open source software) was used for further analysis. Purging the dataset from chimeric reads was performed using USEARCH, while the usearch61 method was used for Operational Taxonomic Units (OTUs) selection. The Greengenes (version 12.10) 16S rRNA gene database was used as reference.

### Statistical methods

DGGE fingerprints were transformed, normalized and analyzed using Bionumerics 7.1 (Applied Maths NV, Belgium) using multidimensional scaling, Principal Component Analysis (PCA), as previously described [17].

Statistical analysis were done using Stata statistical software version 11.2 (SataCorp LP, Texas, USA); two-sample t-test (student’s t-test) was done for numeric data and, in case of non-normally distributed data, data was log transformed.

#### 16 S rRNA gene tag-encoded amplicon sequencing

For calculation of alpha and beta diversity measures, the d- and e-values were set to 11.000 reads per sample (85% of the sequence number of the most indigent sample). Alpha diversity measures expressed with an observed species (sequence similarity 97% OTUs) value were computed for rarefied OTU tables using the alpha rarefaction workflow. Differences in alpha diversity were determined using a t-test-based approach employing the non-parametric (Monte Carlo) method (999 permutations) implemented in the compare alpha diversity workflow. Analysis of similarities (ANOSIM) was used to evaluate group differences using weighted and unweighted UniFrac distance matrices that were generated based on rarefied OTU tables. The relative distribution of the genera registered was calculated and summarized at the genus level OTU tables, followed by Principal Coordinate Analysis (PCoA) plots generated with the Jackknifed beta diversity workflow based on 10 distance matrices calculated using 10 subsampled OTU tables. G-test and ANOVA testing between all sample pairs to test significant differences in beta diversity were conducted using QIIME and multiple (999) rarefied summarized OTU tables [19–21].

## Results

**Fig 1:**
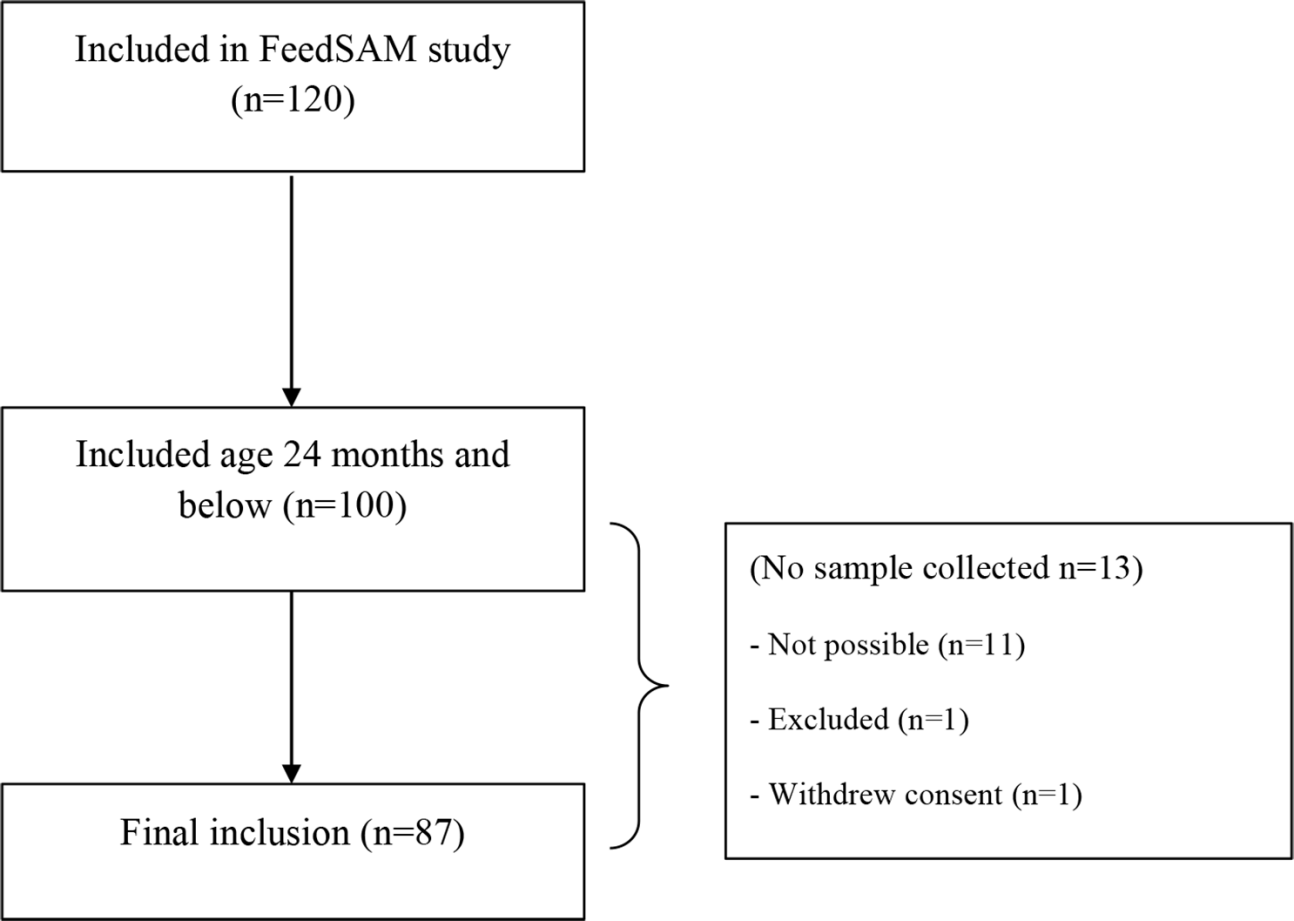
Enrollment. Flow diagram of the final number of children included in the study.

Fecal samples were collected from a total of 87 children aged 6–24 months (Fig 1). Of these 66% were boys, the mean age was 16.1 months, 62% was affected by oedematous malnutrition and 38% by non-oedematous malnutrition. Table 1 provides a detailed description of the two groups of children. As previously reported [22], the oedematous children were slightly older, had higher anthropometric measurements and a higher proportion had skin affections, while a lower proportion had HIV-infection and were breastfed. Other data from the full cohort of children has been published elsewhere [22].

**Table 1:** Characteristics of the 87 severely malnourished children (ntotal = 87; numbers in brackets indicate number of children positive for a given category).

	N^c^	Oedema^a^ (n = 54)	No oedema^a^ (n = 33)	p^b^
Male gender	87	69 (37)	61 (20)	0.45
Age, months	87	17.1 (13.5;20.1)	15.0 (11.8;17.6)	0.04
HIV positive	77	6 (3)	33 (10)	0.004
Diarrhea	82	43 (22)	58 (18)	0.19
Skin affection	85	70 (38)	35 (11)	0.002
Currently breastfed	83	10 (5)	30 (10)	0.02
**Anthropometry**				
Mid-upper arm circumference, cm	87	12.3 +/- 1.3	10.7 +/- 0.7	<0.0001
Weight-for-height, Z^d^	87	-2.9 +/- 1.3	-4.2 +/- 1.0	<0.0001
Height-for-age, Z	87	-2.8 +/- 1.3	-3.1 +/- 1.5	0.36
**Food eaten during last two weeks**^**e**^				
Fish	63	57 (20)	79 (22)	0.25
Nuts	71	85 (34)	84 (26)	0.39
Eggs	62	84 (32)	50 (12)	0.01
Meat	65	74 (29)	69 (18)	0.39
Dairy	74	74 (32)	90 (28)	0.11
Vegetables	67	63 (25)	74 (20)	0.08
Fruit	64	86 (32)	93 (25)	0.55

^a^Values presented are % (n), median (25%;75%) or mean +/- SD

^b^For categorical data, Pearson’s/Fisher’s test was performed, for numeric data, student’s t-test was performed, log-transformed in case of non-normal data

N^c^ = number of children for whom information is available

^d^WHZ were based on the lowest weight recorded (after loss of oedema), for all children

^e^Only caretakers who stayed with the child during the last two weeks were asked.

### Denaturing Gradient Gel Electrophoresis (DGGE)

Significant differences on PC1-values were observed between the two groups of children (p = 0.0317) (S1 Fig). When performing regression analysis of PC1 adjusted for gender and diarrhea at admission, the difference between oedematous and non-oedematous children remained significant (p = 0.032, overall differences among groups p = 0.0018). PC2 and PC3 comparisons were not significant (p = 0.8962 and p = 0.1394).

### 16S rRNA gene tag-encoded amplicon sequencing

We further examined GM composition by tag-encoded 16S rRNA gene amplicon high throughput sequencing. The number of sequences that met all requirements was 7054452 with an average of 81085 sequences (minimum = 13617, maximum = 342628, SD = 52637) per sample, with a mean sequence length of 402 ± 80 bp (study accession number PRJEB10006, sample accession numbers ERS799842-ERS799928).

**Fig 2:**
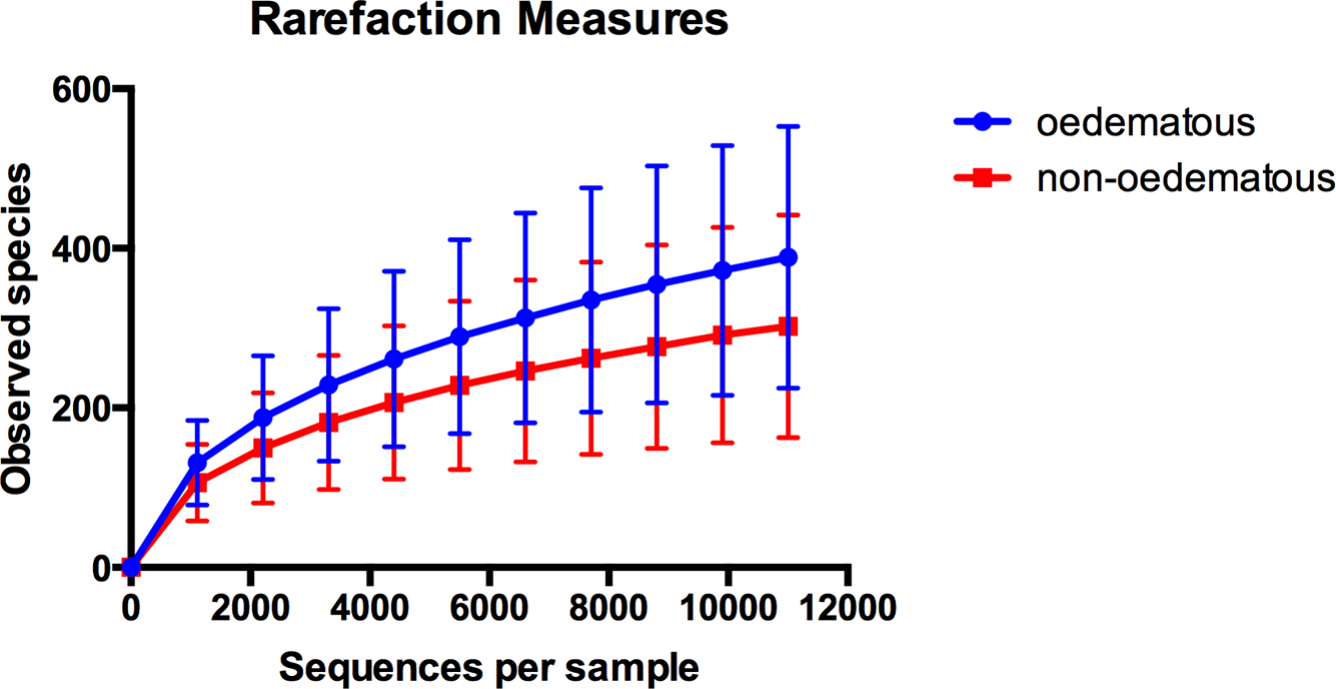
Alpha diversity. Observed species in the GM of oedematous (blue, mean = 378 ± 164) and non-oedematous (red, mean = 305 ± 143) SAM-children (t = 2.0852, p = 0.036).

**Fig 3:**
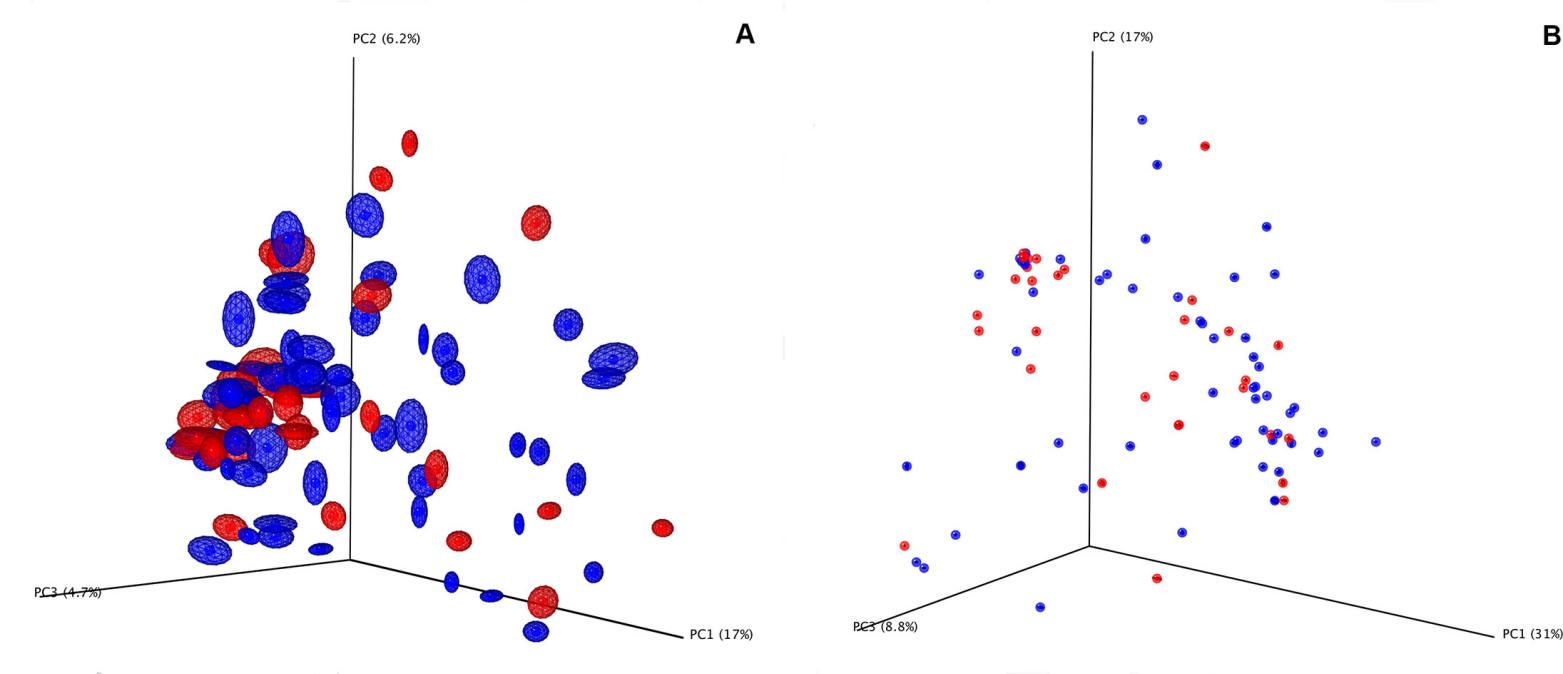
Beta diversity. Principal Coordinate Analysis plot of the tag-encoded 16S rRNA gene amplicon sequencing GM-characterization; A. Unweighted Unifrac distance metrics. Oedematous (blue) vs non-oedematous (red) SAM children (ANOSIM, R = 0.0719, p = 0.011), B. Weighted Unifrac distance metrics. Oedematous (blue) vs non-oedematous (red) SAM children (ANOSIM, R = -0.0085, p = 0.584).

A significant difference in observed species was identified between the oedematous children having a mean of 378 ± 164, and the non-oedematous having a mean of 305 ± 143 observed species (alpha diversity t = 2.0852 and p = 0.036, Fig 2). PCoA and the overall pattern of OTUs, identified by Analysis of Similarities (ANOSIM, beta diversity) revealed a minor significant difference based on unweighted Unifrac analysis (R = 0.0719, p = 0.011) (Fig 3A), while no difference was found based on weighted Unifrac analysis (R = -0.0085, p = 0.584) (Fig 3B). Differences in sampling time after admission could be a confounding factor, but ANOSIM analysis showed no differences between samples obtained within 12 hours of admission and those obtained later (unweighted Unifrac analysis, R = -0.0046, p = 0.517; weighted Unifrac analysis, R = 0.0027, p = 0.404). To investigate the influence of age on GM, we divided the children into age groups 6–12 months and 12–24 months. A minor, but significant influence of age was found based on unweighted Unifrac analysis (R = 0.0895, p = 0.022, S1 Table), while no difference was found based on weighted Unifrac analysis (R = -0.0479, p = 0.834, S1 Table). Severity of wasting was determined by Weight-for Height Z-score (WHZ, below -3 SD), mid-upper arm circumference (MUAC, below 11,5 cm) and Height-for-Age Z-score (HAZ, below -3 SD) did not correlate with GM composition as determined by both unweighted and weighted Unifrac analysis (p>0.05 in all cases, S1 Table). Neither did diet (Table 1) correlate with GM composition (p>0.05 in all cases, S1 Table).

### Abundance and independence of bacterial phyla and genera

The retrieved sequences were distributed between 6 bacterial phyla and 34 genera. The mean relative abundance of the 6 observed phyla did not significantly differ between the non-oedematous (n = 33) and the oedematous (n = 54) children (Table 2). The most abundant phyla in both groups of children were Proteobacteria (mean relative abundance 36% among the oedematous and 50% among the non-oedematous children), Bacteroidetes (35% and 24% respectively) and Firmicutes (24% and 24% respectively) (Table 2). The majority of the Proteobacteria belonged to the Enterobacteriaceae family (28% and 46%, respectively, S2 Table).

**Table 2:** Phyla distribution and abundance, children with oedematous vs non-oedematous SAM.

Phylum	Mean relative abundance (oedema)	Mean relative abundance (non-oedema)	p-value	Bonferroni corrected	FDR corrected
Proteobacteria	36	50	0.0682	0.4094	0.1365
Bacteroidetes	35	24	0.0593	0.3555	0.1778
Firmicutes	24	24	0.9119	5.4712	0.9119
Fusobacteria	2	<1	0.3457	2.0744	0.5186
Actinobacteria	1	1	0.4686	2.8115	0.5623
Unassigned;other	<1	<1	0.0570	0.3421	0.3421

Genera are listed according to abundance in S1 Table. *Prevotella* was the most abundant genus among the oedematous children (mean relative abundance 26%, S2 Table) with no significant difference to the non-oedematous children (mean relative abundance 17%, S2 Table). An unassigned member of the Enterobacteriaceae family was also highly abundant in both groups (24% in the oedematous children, vs. 41% in the non-oedematous children, p>0.05 after Bonferroni/FDR correction for multiple comparisons. No significant differences in the abundance of specific genera were observed after correcting p-value for multiple comparisons (S2 Table). Neither did G-test of independence reveal specific phyla or genera being more associated with either oedematous or non-oedematous SAM (S2 Table).

## Discussion

Our study represents a detailed look at GM composition in a larger cohort of oedematous and non-oedematous SAM children using DNA fingerprinting and high throughput sequencing technologies. Our data shows that non-oedematous SAM children have lower GM alpha diversity compared to oedematous SAM children. However, no clear GM compositional differences were identified between the two groups of children. This indicates that low diversity per se, rather than presence or absence of specific GM members is associated with non-oedematous SAM.

Proteobacteria was the most dominant phyla in both children with oedematous and non-oedematous SAM which is consistent with previous literature [9, 10]. This was primarily reflected in high abundance of Enterobacteriaceae, being abundant in both groups of children. Enterobacteriaceae comprises typical pathogenic intestinal bacteria, such as *Salmonella*, *Yersinia*, *Vibrio* and *Shigella*, but also includes many commensal genera. *Prevotella* which was found to be highly abundant in both groups of children has previously been found to be a predominant GM member in children in Burkino Faso, while it was shown to be absent in age-matched European children [23].

In the same cohort of children, we recently reported breastfeeding to be less frequent in oedematous children, and also that HIV-infection was more frequently reported in non-oedematous children [22]. It is plausible that gut microbiota could be involved in the association of these factors with oedema/non-oedema, but whether a causal relationship exists, remains to be established.

So far, only one previous study has investigated the GM composition of children with oedematous SAM. The GM associated with non-oedematous SAM has not been investigated previously. Smith et al. suggested that *Bilophila wadsworthia* could play a role in the development of kwashiorkor (oedematous SAM), causing a disruptive sulfate metabolism [7]. However, in the present study, *Bilophila* was not detected in any of the two groups of SAM children (S1 Table). However, there is considerable genetic redundancy in the human GM, and changes in the abundance of other GM members involved in sulfate metabolism might still assert influence.

The present study is based on fecal samples, which might not reflect the composition of microbes at gut luminal surface. GM composition at luminal surface might be more determining for the conditions under investigation. This has already been indicated in a recent study focusing on gut microbiota differences between HIV-positive and healthy individuals, showing greater GM differences in endoscopic samples taken directly from the colon and ileum than feces [24]. However, it may not be ethically, nor practically possible to perform endoscopic biopsies in this group of children.

Other factors may also have influenced the GM composition. A large fraction of the stool samples were collected more than 12 hours after admission and thereby after initiation of antibiotic treatment and therapeutic feeding with F75. This is certainly a limitation of the study, but also reflects the difficult practical circumstances under which the samples were obtained. Further, the effect (if any) is minor, as we did not observe any differences in GM composition between samples obtained within 12 hours after admission vs. samples obtained more than 12 hours after admission. Short-term storage at 4°C and -20°C before being moved to -80°C, as done in the present study, has in previous studies not been found to influence DNA quality and down-stream analysis (e.g. high throughput sequencing) [25], but it cannot be ruled out that the sample collection method does have some influence on the results obtained. Sequencing depth is another limiting factor. Here, 13617 high-quality reads or more were obtained per sample. It cannot be ruled out, that even deeper sequencing could have revealed even more details of the GM of SAM children, and further, had deep metagenome sequencing been used for GM characterisation instead of 16S rRNA gene amplicon sequencing, it is possible that differences in the functional capacities between the GM of oedematous and non-oedematous SAM children had been identified. Finally, a comparison between the GM of malnourished children with well-nourished children from the same setting would have been interesting, but unfortunately, specimens enabling such analysis were not obtained.

Furthermore, our results may not be generalizable to children with uncomplicated SAM treated in the community, in other age groups, from different environments and with different HIV prevalences etc.

Although pre- and probiotics have proved to be beneficial for some conditions such as infectious diarrhea [26], only few studies have been conducted testing these functional food components in terms of SAM among children in developing countries [27]. We believe that our finding of GM diversity differences could justify further research in this field of therapeutic strategies.

In conclusion, in this detailed look at GM composition in children suffering from oedematous and non-oedematous SAM, it was found that non-oedematous SAM children have lower GM diversity compared to oedematous SAM children, but no clear compositional differences were found between the two groups of children. Our results may contribute to better understanding of SAM, and inspire for future research of better therapeutic strategies.

## Supporting Information

S1 Checklist STROBE checklist. (PDF)

S1 FeedSAM research protocol Original research protocol of Mwanamugimu study. (PDF)

S1 Table Influence of age, height of age (6–12 months vs. 12–24 months), Weight-for-Height Z-score (WHZ, below -3 SD), Height-for-Age Z-score (HAZ, below -3 SD), mid-upper arm circumference (MUAC, below -3 SD), and influence of different dietary components (eaten/not eaten, see Table 1) on gut microbiota composition in all severe acute malnourished (SAM) children, children with oedematous and non-oedematous SAM. Analysis of Similarities (ANOSIM, beta diversity) based on unweighted and weighted Unifrac distance matrix analysis. Analysis of influence of dietary components only carried out for all children, due to only few individuals reporting intake of some components (Table 1).
(DOCX)

S2 Table Bacterial distribution, abundance and G-test of independence. Children with oedematous vs non-oedematous SAM.
(XLSX)

S1 Fig Multidimensional scaling (PCA) of DGGE-based bacterial fingerprints (V3-region of the 16S rRNA gene) of the oedematous and non-oedematous SAM children. Average +/- SEM PC1 coordinate is plotted against oedematous and non-oedematous SAM children. Difference on PC1 was significant, *p = 0.0317, student’s t-test.
(TIFF)
