# Supplementary material for: Gut Microbiota in Children Hospitalized with Oedematous and Non-Oedematous Severe Acute Malnutrition in Uganda
Source: PLoS Negl Trop Dis. 2016 Jan 15;10(1):e0004369. doi: 10.1371/journal.pntd.0004369 (PMC4714756; doi:10.1371/journal.pntd.0004369)
Supplement: S1 Checklist — (PDF) [file pntd.0004369.s001.pdf]

STROBE Statement—Checklist of items that should be included in reports of ***cross-sectional studies***

	Item No	Recommendation
**Title and abstract**	1	(*a*) Indicate the study's design with a commonly used term in the title or the abstract ***(page 3; the study was nested in a large observational study. Original research protocol uploaded)***
		(*b*) Provide in the abstract an informative and balanced summary of what was done and what was found ***(page 3; collection of fecal samples, DNA-extraction and PCR amplification followed by DGGE-analysis and 16S rRNA gene-targeted high throughput amplicon sequencing. Gut microbiota diversity was found to be lower among non-oedematous compared to oedematous SAM children)***
**Introduction**		
Background/rationale	2	Explain the scientific background and rationale for the investigation being reported ***(page 6; the clinical phenotypes of oedematous and non-oedematous SAM remain unexplained. Recently, malnutrition has been linked to gut microbiota, specifically oedematous malnutrition, kwashiorkor)***
Objectives	3	State specific objectives, including any prespecified hypotheses ***(page 6; we hypothesized that GM composition differs between the two clinical types of SAM, suggesting a possible correlation between GM and the development of the two phenotypes)***
**Methods**		
Study design	4	Present key elements of study design early in the paper ***(page 7 and see item no 1)***
Setting	5	Describe the setting, locations, and relevant dates, including periods of recruitment, exposure, follow-up, and data collection ***(page 7; recruiting of SAM children from Mwanamugimu Nutrition Unit, Mulago Hospital, Uganda, october 2012 to march 2013. For the present study data collection was done at admission and no follow-up was done. For furher information about the main study, see attached original research protocol)***
Participants	6	(*a*) Give the eligibility criteria, and the sources and methods of selection of participants ***(page 7; age 6-24 months, SAM defined as WHZ < -3 SD, MUAC < 11,5 cm and/or bilateral pitting oedema (WHO criteria). Residence close to hospital (for main study). Exclusion criteria were shock, severe respiratory insufficiency, severe bleeding, very severe anemia, weight < 4,5 kg, previous admission in the last 6 months, congenital syndromes and malignancies)***
Variables	7	Clearly define all outcomes, exposures, predictors, potential confounders, and effect modifiers. Give diagnostic criteria, if applicable ***(page 7; the children were grouped according to WHO criteria as oedematous or non-oedematous SAM children, se item 6).***

Data sources/ measurement	8*	For each variable of interest, give sources of data and details of methods of assessment (measurement). Describe comparability of assessment methods if there is more than one group ***(page 7-8; anthropometry and physical examination were performed at admission - we refer to the original research protocol uploaded. Collection of fecal samples, DNA extraction, DGGE followed by 16S rRNA gene tag-encoded amplicon sequencing)***
Bias	9	Describe any efforts to address potential sources of bias ***(to secure consistency, clinical examination was performed by the same medical doctor. Collection of fecal samples was done using the same method for each child)***
Study size	10	Explain how the study size was arrived at ***(page 7; n=87 out of the 120 children included in the main study because of another age grouping. Age 6-24 months were chosen instead of 6-59 months due to theories about final maturation of gut flora during a child's first 2 years)***
Quantitative variables	11	Explain how quantitative variables were handled in the analyses. If applicable, describe which groupings were chosen and why ***(page 6; grouping into oedematous and non-oedematous SAM children, theorizing a possible correlation between gut microbiota and the two clinical phenotypes of SAM)***
Statistical methods	12	(*a*) Describe all statistical methods, including those used to control for confounding ***(page 11; PC1-3 comparisons were done using two-sample t-test for numeric data. In case of non-normally distributed data, a log transformation or Wilcoxon Mann-Whitney U test was performed. Sequencing data ANOVA with Bonferroni correction, g-Test, ANOSIM, $p<0.05$)***
		(*b*) Describe any methods used to examine subgroups and interactions **-** ***not applicable***
		(*c*) Explain how missing data were addressed **-** ***not applicable***
		(*d*) If applicable, describe analytical methods taking account of sampling strategy **-** ***not applicable***
		(*e*) Describe any sensitivity analyses **-** ***not applicable***
**Results**		
Participants	13*	(a) Report numbers of individuals at each stage of study—eg numbers potentially eligible, examined for eligibility, confirmed eligible, included in the study, completing follow-up, and analysed ***(page 12; 87 children eligible and included. Follow up not applicable for this study)***
		(b) Give reasons for non-participation at each stage ***- not applicable***
		(c) Consider use of a flow diagram ***(page; 12, fig. 1)***

Descriptive data	14*	(a) Give characteristics of study participants (eg demographic, clinical, social) and information on exposures and potential confounders ***(page 12; baseline characteristics of included children also showed in table 1. Furthermore, see related published paper from main study attached; Rytter MJ, Namusoke H, Babirekere-Iriso E, Kaestel P, Girma T, Christensen VB, et al. Social, dietary and clinical correlates of oedema in children with severe acute malnutrition: a cross-sectional study. BMC pediatrics. 2015;15(1):25)***
		(b) Indicate number of participants with missing data for each variable of interest ***- (page 12; flow diagram, fig. 1)***
Outcome data	15*	Report numbers of outcome events or summary measures ***(see item 16)***
Main results	16	(*a*) Give unadjusted estimates and, if applicable, confounder-adjusted estimates and their precision (eg, 95% confidence interval). Make clear which confounders were adjusted for and why they were included ***(page 12; DGGE - PC1 comparison was significant different (p=0.032) between the two groups of children. When adjusting for gender and diarrhea at admission, PC1 comparison remained significant different. PC2 and PC3 comparisons were not significant. 16S rRNA gene tag-encoded amplicon sequenceng - a significant difference in observed species was identified (alpha diversity, t=2.0852, p=0.036). A minor significant difference in PCoA and overall pattern of OTUs was found based on unweighted Unifrac analysis (R=0.0719, p=0.011) while no difference was found based on weighted Unifrac analysis (R=-0.0085, p=0.584). No differences were found in terms of phyla and genera mean relative abundance and independence analysis (g-testing))***
		(*b*) Report category boundaries when continuous variables were categorized ***- not applicable***
		(*c*) If relevant, consider translating estimates of relative risk into absolute risk for a meaningful time period ***- not applicable***
Other analyses	17	Report other analyses done—eg analyses of subgroups and interactions, and sensitivity analyses ***- not applicable***
**Discussion**		
Key results	18	Summarise key results with reference to study objectives ***(page 14; a lower gut microbiota diversity was found among non-oedematous SAM children. No clear gut microbiota compositional differences were identified between the two groups of children)***
Limitations	19	Discuss limitations of the study, taking into account sources of potential bias or imprecision. Discuss both direction and magnitude of any potential bias ***(page 15; the study is based on fecal samples which might not reflect the composition of microbes at the gut luminal surface. Gut microbiota composition at luminal surface might be more determining for the conditions under investigation)***

Interpretation	20	Give a cautious overall interpretation of results considering objectives, limitations, multiplicity of analyses, results from similar studies, and other relevant evidence ***(page 15; the study represents a first look at gut microbiota composition in oedematous and non-oedematous malnutrition, comparing the two conditions. Bearing in mind other recent studies linking malnutrition and gut microbiota and the lack of studies testing pre- and probiotics in terms of SAM, we believe our results may contribute to better understanding of SAM and inspire for future research of better therapeutic strategies)***
Generalisability	21	Discuss the generalisability (external validity) of the study results ***(see item 20, improve knowledge about malnutrition, page 14-15; even though our study did not detect bilophila, sulfate metabolism might still influence. We discuss that our results may not reflect SAM children not hospitalized, in different age groups and in different environments with different HIV prevalences)***
**Other information**		
Funding	22	Give the source of funding and the role of the funders for the present study and, if applicable, for the original study on which the present article is based ***(Support was given from the Augustinus Foundation (HF), Brødrene Hartmanns Foundation (MJHR), Arvid Nilssons Foundation (HF), Axel Muusfeldts Foundation (HF), Aase and Einar Danielsens Foundation (MJHR), Torkild Steenbecks Legat (HF), Knud Højgaards Foundation (KHSK), Oticon Foundation (KHSK) and the Danish Free Research Council (MW). The sponsors of the study had no role in the study design, data collection, data analysis, data interpretation, writing of the report, or decision to submit for publication)***

*Give information separately for exposed and unexposed groups.

Note: An Explanation and Elaboration article discusses each checklist item and gives methodological background and published examples of transparent reporting. The STROBE checklist is best used in conjunction with this article (freely available on the Web sites of PLoS Medicine at http://www.plosmedicine.org/, Annals of Internal Medicine at http://www.annals.org/, and Epidemiology at http://www.epidem.com/). Information on the STROBE Initiative is available at www.strobe-statement.org.
