# Supplementary material for: Gut Microbiota in Children Hospitalized with Oedematous and Non-Oedematous Severe Acute Malnutrition in Uganda
Source: PLoS Negl Trop Dis. 2016 Jan 15;10(1):e0004369. doi: 10.1371/journal.pntd.0004369 (PMC4714756; doi:10.1371/journal.pntd.0004369)
Supplement: S1 FeedSAM research protocol — (PDF) [file pntd.0004369.s002.pdf]

Research Proposal:

Re-feeding of severely acute malnourished children at Mwanamugimu Nutrition Unit – Mulago Hospital Complex

Principal Investigator:

Dr. Hanifa Namusoke, MSc, PhD, Senior Nutritionist at Mwanamugimu Nutrition Unit, Department of Paediatrics and Child Health, P.O Box 7072, Kampala, Uganda
Email: n6471277@gmail.com Telephone: 0784 422 714 or 0704 702975
And
Prof Henrik Friis, MD, PhD Department of Human Nutrition, University of Copenhagen, Rolighedsvej 25, 1958 Frederiksberg C, Denmark
Email: hfr@life.ku.dk

Co-investigators

Dr. Elisabeth Kiboneka, Pediatrician, Head of Department*, bethkiboneka@yahoo.co.uk
Dr. Esther Babireke, Pediatrician, PhD student* ebabirekere@yahoo.com
Dr. Ezekiel Mupere, MD, PhD, Senior Lecturer mupez@yahoo.com
Dr. Maren Rytter, MD, PhD student**, mryt@life.ku.dk
Dr. Christian Fabiansen, MD, PhD student, ** chfa@life.ku.dk
Prof. Christian Mølgaard, MD, **, cm@life.ku.dk
Prof. Kim Fleischer Michaelsen, MD, DMSc**, Head of Resarch, kfm@life.ku.dk
Dr. Tsinuel Girma , MD, Pediatrician, Ass. Professor*** tsinuel@yahoo.com
* from Mwanamugimu Nutrition Unit, Department of Pediatrics and Child Health, Mulago Hospital, Kampala, Uganda
** from Department of Human Nutrition, University of Copenhagen.
*** Jimma University Specialized Hospital, Ethiopia

Introduction

Children hospitalized for severe acute malnutrition (SAM) are very vulnerable. Although treatment protocols have improved during recent years, many centers in the world still report unacceptably high mortality rates among hospitalized children with severe acute malnutrition. Particularly the children who develop oedematous malnutrition seem to be at high risk of dying, with the etiology of this condition remaining essentially obscure.

Some is known about the reasons for this high mortality - and a lot is unknown. In order to understand why these children die, and be able to provide better treatment in the future, we need to understand the physiological changes taking place during recovery from severe acute malnutrition.

With this purpose, we wish to follow a group of children admitted to the Mwanamugimu Nutrition Unit during their treatment and recovery. We will assess their balance of salt and nutrients in the blood, their immune system, intestinal function and heart function. We will follow them up after discharge to

see how they grow and develop. And we hope to understand why some children develop oedema with malnutrition and why they are so particularly vulnerable.

Objective

To assess changes in physiological parameters in malnourished children at admission, during treatment and after recovery, with the aim of identifying areas where treatment can be improved. This includes changes in immune function, morbidity, electrolyte homeostasis, growth and development, and to identity pathophysiological features of edematous malnutrition.

Background and literature review

Hospitalized children with severe acute malnutrition (SAM) are at high risk of dying. Although outcome of treatment has improved with the emergence of community based management and the development of protocols for in-patient treatment, many centres in the world still report unacceptably high mortality rates among hospitalised children with SAM[1]. Currently, the mortality rate at Mwanamugimu, a nutrition rehabilitation unit at Mulago Hospital, Kampala, is estimated to be around 18%[2]. In other centres in low-income countries, mortality can be even higher[1]. Several reasons have been suggested for this.

First, hospitalized children represent the most sick of all malnourished children, often with co-morbidities, especially HIV and severe systemic infections[3 4]. Second, malnourished children have altered physiology compared to normal children: their cardio-vascular system is impaired, rendering them susceptible to fluid overload and cardiac failure, and their immune system is deficient, making them susceptible to severe systemic infections. And third, complications occur during treatment, as electrolyte disturbances and malabsorption. Finally, we still do not understand well the changes that take place in malnourished children. Particularly, we do not know the aetiology and pathogenesis of oedematous malnutrition[5]. Thus, treatment of severely malnourished children has room for improvement.

To offer better treatment we need a better understanding of the physiology of SAM before and during treatment. Four areas are of particular interest:

a) Electrolyte disturbances.

b) Infection, immunity, inflammation and gut permeability

c) Myocardial function

d) Child development and long term outcome

Electrolyte disturbances

Malnourished children often develop electrolyte disturbances, and electrolytes may become further disturbed upon re-feeding. During negative energy balance, the body stores of phosphosous (P), potassium (K) and magnesium (Mg) are diminished. However, plasma levels remain within the normal range due to compensatory mechanisms. Upon re-feeding, insulin levels rise, causing a shift of glucose from the extracellular space into the intracellular space, with K, P and Mg following. Furthermore, the synthesis of protein, fat and glycogen increase requirements for the nutrients and contributes to nutrient depletion[6]. This condition has been termed “refeeding syndrome”. Unless the nutrients are supplied in sufficient amounts, the depletion can result in severe hypo-phosphataemia, hypo-kalaemia, hypo-magnesiaemia, which can cause heart failure or even death[6]. Awareness of this condition in the treatment of anorexia nervosa, have allowed a more aggressive nutritional therapy and resulted in a

better prognosis. In a few studies, severe hypophosphatemia has been reported in a number of children during treatment of SAM with oedema[4]. This suggests that re-feeding syndrome does occur in children with SAM, even during optimal nutritional rehabilitation. But the extent to which it occurs, and whether it can be prevented by increasing the amount of phosphate in the nutritional therapy is still unknown.

Infection, immunity, inflammation and gut permeability

It is generally accepted that malnourished children have impairment of immune functions and are more susceptible to infectious diseases [7]. The immunodeficiency of malnutrition is considered a major reason for the high incidence of systemic infections in severely malnourished children[8] . However, the precise mechanism behind this "nutritionally acquired immunodeficiency" is not well understood.

Malnourished children have thymic atrophy[9]. The thymus regains its size with nutrition rehabilitation, but at a slower rate than weigt gain[10]. Children with malnutrition also have cell-mediated anergy measured by no or diminished delayed type hyper-sensitivity reactions[11], reduced secretory IgA (sIgA) is reduced in saliva and tears[12], and diminished levels of complement C3 in blood[13].

In contrast to this, other parts of the immune system seem remarkably intact with malnutrition. Differences in number of white blood cells, T-cells and B-cells in peripheral blood have not been identified[14] [15], and humoral immunity seems unaffected by malnutrition[16].

Traditionally, the immunodeficiency of malnutrition has been explained by failure to provide fuel for the energy-demanding immune-response[17]. However, this does not explain why some parts of the immune system are intact even with severe malnutrition. Recent studies of malnourished mice have found that while production of pro-inflammatory cytokines is reduced, production of anti-inflammatory cytokines seems sustained. This has led to the "tolerance hypothesis", that the immunodeficiency seen in malnutrition could be a *regulated* process, aiming at preventing auto-immune reactions resulting from release of self-antigens from catabolism and cellular death[18]. If this is the case, the susceptibility to infectious diseases may be the price of preventing autoimmune inflammatory reactions during malnutrition.

Perhaps oedematous malnutrition could represent a condition of dys-regulated inflammation resulting from catabolism and insufficient down-regulation of the inflammatory responses. To assess whether this is the case, a central feature to assess is immune regulatory mechanisms such as T cell subsets with pro- and anti-inflammatory properties. Regulatory T cells (Tregs) are anti-inflammatory, while Th17 cells have pro-inflammatory properties [19]. While Tregs inhibit autoimmunity, Th17 cells play a role in the induction of autoimmune tissue injury.

The balance between Tregs and Th-17 cells is essential and may give a clue as to whether the immune system is "tuned" towards tolerance or reactivity. Increased levels of Tregs in children with malnutrition would support the "tolerance hypothesis" with immunodeficiency of malnutrition being caused by a regulated process aimed at preventing autoimmune disease by down-regulating immune activation. If oedematous malnutrition indeed should represent an inflammatory reaction to catabolism, it would most likely result from a failure to mount Tregs.

Many children with malnutrition have enteropathy[20] [21] with increased gut permeability, providing an entry for toxins like Lipopolysaccharide (LPS) from the gut into the bloodstream[22]. Possibly, the resulting inflammatory reactions may stimulate the immune system and play a role in the development of of Kwashiorkor.

Myocardial function

Malnutrition may lead to cardiac abnormalities. The loss of heart muscle combined with fluid-electrolyte disturbances can cause hypotension, arrhythmias, cardiac failure and even death. A number

of investigations can evaluate the state and function of the heart: *Echocardiography* (cardiac ultrasound) is used extensively. The cardiac biomarkers troponin (TN) and natriuretic peptides can be measured in plasma. TN is known to be diagnostic for myocyte damage also in pediatric malnutrition[23]. The cardiac hormones Atrial Natriuretic Peptide (ANP) and Brain-type Natriuretic Peptide (BNP) are elevated in cardiac hypertrophy and heart failure [24]. However,
how these peptides are related to degree and type of malnutrition, and if they have prognostic value for outcome is still unknown. ECG is a non-invasive investigation used extensively in diagnosis, management, and follow-up of patients with any suspected or known heart diseases. Electrodes are placed on the outside of the body and an ECK is recorded fast causing no pain and minimal discomfort in the placement of electrodes.

Long term outcome and child development

Few studies have assessed the long term outcome after admission with severe acute malnutrition. It is known, that children with SAM often have developmental delay when they are malnourished, and child stimulation and play is an important part of the WHO protocol of management of SAM. It is not clear how development is affected by having survived an episode of SAM or whether a "developmental catch-up" takes place after recovery. Child development is related to the level of physical activity, and lack of physical activity may be the link between malnutrition and developmental impairment. Furthermore, very little is known about the status of essential fatty acids in malnourished children, which is also thought to be crucial for normal child development and intellectual abilities[25].

Site: Mwanamugimu Nutrition Rehabilitation Unit

The study will be conducted at Mwanamugimu Nutrition Unit (MNU) in the Department of Paediatrics and Child Health, Mulago National Referral Hospital. Established in 1964, and recently restored to a center of excellence, it serves as a national referral facility for management of acute malnutrition in Uganda. The unit provides In-patient Therapeutic Care, where patients with SAM and medical complications are treated and Out Patient Therapeutic care (OTC), treating uncomplicated severely and moderately malnourished children and patients discharged from ITC, using ready to use therapeutic food (RUTF).

Standard treatment protocol

Uganda follows the approach stated in the Integrated Management for Acute Malnutrition. The treatment is based on therapeutic milk diets (F-75 and F-100) to children hospitalized with SAM, and treating medical complications with drugs. A child is considered to have SAM if it has either weight for height (W/H) below the -3 standard deviations (SD) or mid upper arm circumference (MUAC) below the cut-off points for SAM or pitting oedema developing from both feet

If a child also has complications (lack of appetite, vomiting, convulsions, lethargy, hypoglycaemia, high fever, hypothermia, severe dehydration, lower respiratory tract infection, severe anaemia, skin lesion or signs of vitamin A deficiency) he/she is admitted for treatment in two phases.

Phase I: Stabilization

The child receives antibiotics, relevant vitamins and immunization up-dates when necessary. The child is fed cautiously with F-75 in small frequent feeds. Phase I typically lasts three to seven days, until medical complications have been resolved.

Transition phase

The child starts receiving F-100 and continues with relevant drugs. The feeds are increased as the child expresses appetite. A child will remain in *transition phase* for at least 2 days, or until oedema has almost subsided, no naso-gastric tube is needed and there are no serious infections.

Phase II: Rehabilitation
In Phase 2, the child receives F-100 in larger amounts, to gain weight.

Discharge
The child is discharged once it gains of 20% of the admission weight after loss of oedema. The child is then followed up through the Outpatient Therapeutic Center (OTC) and treated with RUTF and relevant drugs, until another 20% weight gain.

Objectives

Development objectives
To improve rehabilitation of children with severe acute malnutrition

Capacity strengthening objectives
To strengthen the research capacity at Mwanamugimu Nutrition Unit

General research objective
To assess changes in physiological parameters in children admitted with severe acute malnutrition at admission, during treatment and after recovery

Specific research objectives

Primary objective:
To assess changes in serum phosphate during treatment of children with SAM with therapeutic milk

Secondary objectives:
- To assess child development and physical activity during treatment
- To assess level and predictors of blood essential fatty acids (EFA) on admission, and changes during treatment
- To assess the role of blood EFA for child development and physical activity
- To assess changes in hemoglobin and serum ferritin during treatment
- To compare levels of regulatory T cells and Th17-positive T-cells (T-cell subsets), inflammatory and anti-inflammatory cytokines as well as thymic size in well-nourished children compared to malnourished children with and without oedema, and relate immune parameters to breast feeding and components in breast milk.
- To assess gut permeability by Lactulose-Mannitol Test (LM-test) and gut flora in children with oedematous malnutrition and children without oedema, compared to well-nourished children and relate this to LPS in blood as well as sIgA in breast milk.
- To assess levels of in Tn, ANP and BNP in relation to myocardial function and serum phosphate in malnourished children.

- To assess long-term outcome by morbidity, anthropometry, child development and thymic size after discharge

Methods

Study design, study area and study population

The study will be a longitudinal observational study among children admitted for in-hospital treatment with SAM at MNU. The children will receive the standard treatment based on the Ugandan national protocol. In addition to standard examinations, a number of assessments and investigations will be carried out, and the children will be seen again for follow-up after discharge.

Recruitment rate:

The unit admits an average of 123 (range 81-190) children per month. With anticipated consent of 50 %, 60 patients are expected to be eligible for inclusion per month. The study will run from September 2012 to November 2012 and include approximately 120 children.

Sample size considerations and data analyses:

Rather than testing a single hypothesis, the study will use an explorative approach to describe the changes in serum phosphate and other parameters during recovery, and the role of various exposure variables such as presence of oedema, HIV, etc. With 120 children in the study, and with 50% with oedema, we will have 90% power to detect a difference of 0.6 SD or more of any normally distributed variable with a 5% significance level. Similarly, with a control group of 20 children, we will have 90% power to detect differences of 0.8 SD or more between children with SAM and control children, of any normally distributed variable. The control group will be crucial, as there are variables for which we have no reference data, hence we need to know the effect of SAM by comparing with the control group.

Inclusion criteria

Children between 6 - 59 months of age admitted to MNU pediatric wards with either:

- W/H < -3 Z-score (based on the WHO growth standard)
- or MUAC <11.5 cm
- or Pedal bilateral pitting oedema
- AND living within 7 km from the study site

Exclusion criteria

- Shock, severe respiratory difficulty or significant bleeding at the time of admission. Once stabilized, these children may be included.
- weight below 4,5 kg at admission or severe anemia

These children are excluded because the study involves drawing of a total of 13 ml of blood, and only by excluding these children can we be sure that this amount of blood is less than or “minimal risk” to the childrens health.

- Severe developmental delays, significant congenital diseases or malignant diseases.
- Within the last six months having been admitted for SAM.

In these children, an underlying disease is likely causing the malnutrition. One reason for excluding them is, that making the pattern of recovery different from that of children with primary malnutrition or malnutrition related to infectious disease, and thus the results less generalizable to other

circumstances. Another reason is that we want to assess their motor, language and social development – which is likely to be affected by their underlying condition, blurring the effect of malnutrition.

Reference group

Because we wish to assess the effect of malnutrition on a number of physiological parameters, and because some of these parameters are characterized by no known normal values, or the values being highly dependent on the geography, on the examiner, or on the method used, a small reference group of normal children is needed for comparison.

20 healthy children of similar age and sex will be invited to participate from the nearby health centres when they come for routine vaccination, provided that their parents give informed consent for participation. This number of children is calculated from the parameters planned to be studied (thymus size, T-cell subsets, gut permeability and fatty acid status). For these parameters 20 children are anticipated to be sufficient to detect a difference.

Time Plan

The children will be recruited for the study as outlined in fig. 1

	Aug 12	Sep 12	Oct 12	Nov 12	Dec 12	Jan 13	Feb 13	Mar 13	Apr 13	May 13
**Pilot**	XXX									
**Inclusion**		XXX	XXX	XXX	XXX	XXX	XXX			
**Follow-up**				XXX	XXX	XXX	XXX	XXX	XX X	XXX
**Sample analysis**						XXX	XXX	XXX	XX X	XXX

Figure 1: Time plan of the study

Data management

Appropriate data quality measures will be taken at different stage of the study. Overall control and monitoring of the study conduct will be conducted by the Senior Nutritionist of Mulago hospital complex and any other trained study personnel. All patient data will be typed into a computerized database in anonymous form showing only study ID number.

Data collection

Most assessments for the study will take place at three times during the stay in hospital: At admission, at start of phase II and at discharge. A few assessments (weight, vital signs, feed intake) will be recorded daily. Some measurements will be repeated at follow-up at week eight.

The following data will be collected from all participating children:

Questionnaire data

At admission, a structured questionnaire will obtain data on socio-demography and feeding history and morbidity.

Milk intake

The amount of therapeutic milk consumed will be recorded throughout the study period. If the child is breastfeeding, the mother will be asked to count approximately how many times the child breastfeeds on day two and at discharge.

Clinical data

Baseline clinical data will be reviewed from the clinical monitoring tool, including diagnoses, laboratory tests performed and treatment. Abnormal clinical signs will be recorded.

Physical examination

Vital signs (Body temperature, pulse and respiratory rate) and degree of oedema will be recorded daily. A clinical examination will be done at admission, at discharge and at follow-up.

Anthropometry

Height, MUAC and knee-heel length will be measured for all children at admission, at start of phase II and at discharge. Weight is measured daily.

Ultra-sound examination:

The size of the thymus and the myocardial function will be assessed by ultrasound on day two, at start of phase II, at discharge and at follow-up at eight weeks after admission.

E*lectrocardiography*

E*lectrocardiography (ECG) will be done* at admission and at discharge for all study participants.

Breast milk samples

If the child is breastfeeding, a sample of 7 ml hind milk will be obtained at admission and analysed for level of sIgA and IL-7.

Blood samples

5 mL of blood will be collected at two different occasions: At admission and at discharge. Of this, 1 ml will be used to measure an inflammatory marker (CRP), markers of kidney function (Creatinin), as well as electrolytes (Phosphate, pH, Ca+, Na, Cl) and albumin. 4 ml will be used to measure levels of different subsets of T-cells as well as levels of anti-inflammatory cytokines, LPS, troponin, ANP and BNP in plasma. A drop of blood will be used for haemoglobin measurement and another drop will be stored on filter paper for analysis of fatty acid status. On additional three days, when moving to a new phase of nutritional treatment and 48 hours after, a small amount (1 ml) of blood will be collected for determination of phosphate, electrolytes and pH. In total, 13 ml of blood will be drawn from each child during the study. At follow-up after 8 and 16 weeks post-admission, a finger-prick blood sample will be collected for determination of fatty acid status

Urine samples

To measure intestinal permeability, the LM-test will be carried out on day two and at discharge. For this, the child will drink a sugar solution of lactulose and mannitol adjusted to body size. Urine will be collected for the next 5 hours and analyzed for lactulose and mannitol. A sample of this urine will be kept for analysis of metabolites.

Faecal sample

If the child presents clinical signs of malabsorption (loose stools, green stools, flatulence) its stool pH will be tested and also for reducing substances. A stool sample (about 1 g) will be collected and examined for microbial flora at admission at start of phase II, at discharge and, if possible at follow-up at 8 weeks after admission.

Physical activity

Physical activity will be assessed by accelerometer (Actigraph GTX3+), a small electronic device carried by the child in an elastic belt for 3-5 consecutive days, just before discharge.

Child development

In a playful way the development of the child will be assessed in the domains of gross motor, fine motor, language and social development, at entry to phase II, at discharge and at follow-up 8 weeks after admission

Follow-up assessment

The children included in the study will be invited to come back for two follow-up assessment after discharge, 8 weeks and 16 weeks after admission. At this point the caretakers will be interviewed about the symptoms and well-being of their child, a full physical examination including antropometry, vital signs and ultrasound of thymus and heart will be carried out, and the development of the child will be assessed in a playfull way. A fingerprick blood sample will be collected for analysis of fatty acid status. If feasible, a stool sample will be obtained. The interval of 8 and 16 weeks is chosen because 8 weeks has previously been shown to be the time for full recovery, not only antropometrically, but also immunologically (measured by thymic size)[26], and 16 weeks will give a picture of development and wellbeing on a longer term. If funding allows it, another follow-up visit will be arranged, in which case a new propsal will be submitted to the IRB.

	Hospital										Home	
	Phase I			Transition			Phase II			Dis-charge	8 weeks	16 weeks
**Assessment, Day**	0	1	2	0	1	2	0	1	3			
Milk intake, Vital signs, Weight	Dail y										X	X
Questionnaire	X										X	X
Medical examination Anthropometry	X									X	X	X
Ultrasound: Thymus,			X				X			X	X	
Ultrasound: Heart ECG	X									X		
Blood sample	5 ml		1 ml	1 ml		1 ml				5 ml	Finger prick	Finger prick
Urine samp. (LM-test)			X							X		
Stool sample	X						X			X	X	
Breast milk sample			X							X		
Physical activity										X		
Child development							X			X	X	X

Figure 3: Overview of assessments.

Data collection in the reference group of healthy children

The assesments done in these children are only those for which no known normal values exist, or for which the result is highly dependent on the examiner, the method used or geography.

These are thymic size (measured by ultrasound), gut permeability (measured by lactulose-mannitol test) and fatty acid status and number of T-cell subsets (measured in a blood sample of 4 ml), all carried out as described above

Data collectors

All measurements and questionnaires will be performed by PhD or Masters students, medical doctors, trained research nutritionists, nurses or health workers. Standard operational procedures (SOP) will be followed for all activities stated.

Laboratory tests

Heamoglobin and CRP will be measured on the local lab of MNU, using bed-side test devices. Theese will be calibrated regularly using test-samples of known values.

Other lab analyses will primarily be conducted by MU-JHU Core Laboratory and Infectious Diseases Institute (IDI) – Mulago hospital complex. This is a research lab supported by John Hopkins University which is accredited by College of American Pathologists (CAP) with currently on-going quality control and certified for quality and GCLP. For quality control, a few samples are planed to be

double-analysed here as well as in a collaborating laboratory in Mwana, Tanzania, which has extensive expertise in measuring phosphate.

Laboratory tests not available in Uganda will be shipped to laboratories abroard for analysis.

The analyses of fatty acids will be done in Canada at University of Waterloo, Department of Kinesiology, a lab which has developed a pioneering method of assessing fatty acid status on a dried blood spot on filter paper.

The analyses of immune parameters (T-cell subsets and cytokines) will be analysed in the Department of Infectous Disease, Copenhagen University Hospital, Hvidovre. This is a certified biosafety level 2 laboratory, with advanced multi-colour flow-cytometry. It has on-going quality control, and is accredited by Joint Commision International.

The analysis of cardiac enzymes will be done at Department of Biochemistry at Rigshospitalet, Copenhagen, the National Referral Hospital of Denmark, which also has current internal and external quality control.

Analysis of lactulose-mannitol ratio and stool microbiota will be done at Department of Human Nutrition, University of Copehagen. This lab has on-going quality control and extensive experience with the mentioned analyses.

Data analysis

The primary outcome of the study is level of and changes in serum phophate during treatment, while the results of the other assesments (child development, immune function, heart function, gut permeability) are secondary outcomes.

Data will be analysed using the intercooled version of the statistical software package Stata version 12 (Stata/IC) (StataCorp LP, College station, Texas, USA)). Baseline values of serum phosphate and other exposures will be presented as mean ±sd and differences between categories of exposure variables will be analyzed by t-tests and one-way analysis of variance. Final multivariable models will be developed with serum phosphate and other baseline characteristics as outcome, and oedema, HIV and other factors as predictors, while controlling for age and sex. To assess the changes over time and the factors affecting it, repeated measures of serum phosphate and other variables will be analyzed using multilevel mixed-effects linear regression model by using xtmixed.

Research capacity strengthening

In addition to conducting important research, strengthening of research capacity is a key objective of this project. As stated in the Memorandum of Understanding between MNU and UC, exchange of staff and students are key components of the collaborative research.

As such, the main paper from this project, addressing the primary objective mentioned above, will be written by Senior Nutritionist, Hanifa Namusoke, PhD, as her post-doc research project. The plan is that Dr. Namusoke will write up the data during a sabbatical stay in Denmark. In addition, Dr. Esther Babireke will get PhD-training with registration at University of Copenhagen. The focus of her PhD will be essential fatty acids and child development. Dr. Ezekiel Mupere will serve as Ugandan co-supervisor. In addition, Drs. Maren Rytter and Christian Fabiansen will be involved and write 1-2 papers based on data from the study. Other students may also be involved.

Ethical considerations

Authorizations

The protocol will be submitted for approval to the ethical committee of the School of Public Health, Makerere University and later to Uganda National Council of Science and technology (UNCST) and to the Danish National Committee on Biomedical Research Ethics. The study will be conducted in accordance with the principles of the Declaration of Helsinki and international ethical guidelines for biomedical research involving human subjects, published by the Council for International Organizations of Medical Sciences (CIOMS).

Informed consent

Parents or guardians of the child will be given detailed verbal and written information about the study and the examinations that will be conducted. The information material will be translated to Luganda and other relevant languages and back-translated to ensure accuracy. After answering any questions about the study, and giving sufficient time to consider, the parent or caretaker will be asked if they wish to participate, and in that case to sign the trial consent form. If the parents are illiterate, a fingerprint will be accepted instead of a signature. In that case, a literate witness is required to be present during the reading of the consent form and sign in addition to the fingerprint from the caretaker.

Only children whose parents provide informed content will be included, and consent can be withdrawn at any time during the study.

Patient information will be kept confidential and not disclosed to anyone outside the study team and clinical staff, unless explicit permission is obtained from the specific caretaker. When study assessments identify an abnormal condition requiring treatment relevant data will be provided to the clinical staff and actions will be taken to facilitate treatment. The database will not contain names or address of patients to ensure their anonymity.

Risks and discomforts

There are no or minimal risks associated with the procedures and examinations carried out in the study. Efforts will be made to make all assessments as considerate as possible and to not disturb the child or caretaker when sleeping, feeding ect.

Blood sampling will be carried out using anaesthetic crème to minimize pain and discomfort. Efforts will be made not to draw more blood than absolutely essential, and the amount drawn (max 7 ml on each occasion and max 14 ml in total from each child) is well within the “safe amounts” recommended for blood sampling for reseach purposes[27] since children with body weights below 4,5 kg and those with severe anaemia will be excluded from the study. The puncture may cause a localized bruise and rarely, infection.

Ultrasonography assessment of thymus size and myocardial function is a safe, non-invasive painless procedure that has previously been used in malnourished children. It takes about 15 minutes and can be carried out with the child on the mothers lap. It does not involve any discomfort. ECK is non-invasive, safe and painless and takes about 3 minutes.

The sugar solution (250 mg/mL Lactulose and 50 mg/mL Mannitol) that the child drinks for the test of intestinal permeability is adjusted to the child’s size (2 ml/kg body weight). The solution is safe but may cause transient loose stools in the hours after consumption. Urine collection is non-invasive and safe, however, small discomfort may be experienced upon removal of the urine bag, similar to removal of a patch.

Benefits for participants

Parents will receive no payments for participating in the study. However, they may benefit from the extra attention and examinations that are part of the study, and all information that is generated will be shared the health staff as soon as available, to optimize treatment. A small gift will be provided to families after participating in the study, and additional costs they may have

References

1. Brewster DR. Inpatient management of severe malnutrition: time for a change in protocol and practice. *Ann Trop Paediatr*. 2011;31(2):97–107.

2. Namusoke H. Treatment of severe acute malutrition. 2011.

3. Bachou H, Tylleskär T, Downing R, Tumwine JK. Severe malnutrition with and without HIV-1 infection in hospitalised children in Kampala, Uganda: differences in clinical features, haematological findings and CD4+ cell counts. *Nutr J*. 2006;5:27.

4. Babirekere-Iriso E, Musoke P, Kekitiinwa A. Bacteraemia in severely malnourished children in an HIV-endemic setting. *Ann Trop Paediatr*. 2006;26(4):319–328.

5. Ahmed T, Rahman S, Cravioto A. Oedematous malnutrition. *Indian J. Med. Res.* 2009;130(5):651–654.

6. Fuentebella J, Kerner JA. Refeeding syndrome. *Pediatr. Clin. North Am.* 2009;56(5):1201–1210.

7. Chisti MJ, Pietroni MAC, Smith JH, Bardhan PK, Salam MA. Predictors of death in under-five children with diarrhoea admitted to a critical care ward in an urban hospital in Bangladesh. *Acta Paediatr.* 2011;100(12):e275–279.

8. Schaible UE, Kaufmann SHE. Malnutrition and infection: complex mechanisms and global impacts. *PLoS Med.* 2007;4(5):e115.

9. Aref GH, Abdel-Aziz A, Elaraby II, et al. A post-mortem study of the thymolymphatic system in protein energy malnutrition. *J Trop Med Hyg*. 1982;85(3):109–114.

10. Chevalier P, Sevilla R, Sejas E, et al. Immune recovery of malnourished children takes longer than nutritional recovery: implications for treatment and discharge. *J. Trop. Pediatr.* 1998;44(5):304–307.

11. McMurray DN, Watson RR, Reyes MA. Effect of renutrition on humoral and cell-mediated immunity in severely malnourished children. *Am. J. Clin. Nutr.* 1981;34(10):2117–2126.

12. Watson RR, McMurray DN, Martin P, Reyes MA. Effect of age, malnutrition and renutrition on free secretory component and IgA in secretions. *Am. J. Clin. Nutr.* 1985;42(2):281–288.

13. Forte WC, Forte AC, Leão RC. Complement system in malnutrition. *Allergol Immunopathol (Madr)*. 1992;20(4):157–160.

14. Hughes SM, Amadi B, Mwiya M, et al. CD4 counts decline despite nutritional recovery in HIV-infected Zambian children with severe malnutrition. *Pediatrics*. 2009;123(2):e347–351.

15. Nájera O, González C, Toledo G, López L, Ortiz R. Flow cytometry study of lymphocyte subsets in malnourished and well-nourished children with bacterial infections. *Clin. Diagn. Lab. Immunol.* 2004;11(3):577–580.

16. Schopfer K, Douglas SD. In vitro studies of lymphocytes from children with kwashiorkor. *Clin. Immunol. Immunopathol.* 1976;5(1):21–30.

17. Chandra RK. Nutrition and the immune system: an introduction. *Am. J. Clin. Nutr.* 1997;66(2): 460S–463S.

18. Monk JM, Richard CL, Woodward B. A non-inflammatory form of immune competence prevails in acute pre-pubescent malnutrition: new evidence based on critical mRNA transcripts in the mouse. *The British Journal of Nutrition*. 2011:1–5.

19. Afzali B, Mitchell P, Lechler RI, John S, Lombardi G. Translational mini-review series on Th17 cells: induction of interleukin-17 production by regulatory T cells. *Clin. Exp. Immunol.* 2010;159(2): 120–130.

20. Gupta SS, Mohammed MH, Ghosh TS, et al. Metagenome of the gut of a malnourished child. *Gut Pathog*. 2011;3:7.

21. Campbell DI, Lunn PG, Elia M. Age-related association of small intestinal mucosal enteropathy with nutritional status in rural Gambian children. *Br. J. Nutr.* 2002;88(5):499–505.

22. Campbell DI, Elia M, Lunn PG. Growth faltering in rural Gambian infants is associated with impaired small intestinal barrier function, leading to endotoxemia and systemic inflammation. *J. Nutr.* 2003;133(5):1332–1338.

23. Faddan NHA, Sayh KIE, Shams H, Badrawy H. Myocardial dysfunction in malnourished children. *Ann Pediatr Cardiol*. 2010;3(2):113–118.

24. Tobias JD. B-type natriuretic peptide: diagnostic and therapeutic applications in infants and children. *J Intensive Care Med*. 2011;26(3):183–195.

25. Lauritzen L, Hansen HS, Jørgensen MH, Michaelsen KF. The essentiality of long chain n-3 fatty acids in relation to development and function of the brain and retina. *Prog. Lipid Res.* 2001;40(1-2):1–94.

26. Chevalier P, Sevilla R, Sejas E, et al. Immune recovery of malnourished children takes longer than nutritional recovery: implications for treatment and discharge. *J. Trop. Pediatr.* 1998;44(5):304–307.

27. Howie SRC. Blood sample volumes in child health research: review of safe limits. *Bull. World Health Organ.* 2011;89(1):46–53.
