# Supplementary material for: Gut Microbiota in Children Hospitalized with Oedematous and Non-Oedematous Severe Acute Malnutrition in Uganda
Source: PLoS Negl Trop Dis. 2016 Jan 15;10(1):e0004369. doi: 10.1371/journal.pntd.0004369 (PMC4714756; doi:10.1371/journal.pntd.0004369)
Supplement: S1 Table — Analysis of Similarities (ANOSIM, beta diversity) based on unweighted and weighted Unifrac distance matrix analysis. Analysis of influence of dietary components only carried out for all children, due to only few individuals reporting intake of some components (Table 1). (DOCX) [file pntd.0004369.s003.docx]

**S1 Table.** Influence of age, height of age (6-12 months *vs.* 12-24 months), Weight-for-Height Z-score (WHZ, below -3 SD), Height-for-Age Z-score (HAZ, below -3 SD), mid-upper arm circumference (MUAC, below 11,5 cm), and influence of different dietary components (eaten/not eaten, see Table 1) on gut microbiota composition in all severe acute malnourished (SAM) children, children with oedematous and non-oedematous SAM. Analysis of Similarities (ANOSIM, beta diversity) based on unweighted and weighted Unifrac distance matrix analysis. Analysis of influence of dietary components only carried out for all children, due to only few individuals reporting intake of some components (Table 1).

|  | All SAM children | | | | Oedematous SAM | | | | Non-oedematous SAM | | | |
| --- | --- | --- | --- | --- | --- | --- | --- | --- | --- | --- | --- | --- |
|  | Unweighted | | Weighted | | Unweighted | | Weighted | | Unweighted | | Weighted | |
| Parameter | R | p | R | p | R | p | R | p | R | p | R | P |
| Age | 0.090 | **0.022** | -0.048 | 0.83 | 0.032 | 0.31 | -0.047 | 0.65 | 0.026 | 0.20 | -0.016 | 0.61 |
| WHZ | -0.013 | 0.68 | -0.021 | 0.76 | -0.035 | 0.93 | -0.002 | 0.49 | -0.097 | 0.74 | -0.12 | 0.72 |
| HAZ | 0.044 | 0.12 | 0.046 | 0.17 | 0.043 | 0.20 | 0.016 | 0.35 | 0.016 | 0.36 | 0.084 | 0.20 |
| MUAC | 0.028 | 0.078 | 0.022 | 0.12 | -0.075 | 0.97 | -0.070 | 0.91 | 0.000 | 0.46 | 0.22 | 0.17 |
| Fish | -0.030 | 0.76 | -0.005 | 0.49 |  |  |  |  |  |  |  |  |
| Meat | 0.066 | 0.089 | -0.047 | 0.78 |  |  |  |  |  |  |  |  |
| Nuts | -0.0004 | 0.48 | -0.053 | 0.73 |  |  |  |  |  |  |  |  |
| Eggs | 0.006 | 0.41 | 0.005 | 0.44 |  |  |  |  |  |  |  |  |
| Dairy prod. | -0.013 | 0.57 | -0.078 | 0.86 |  |  |  |  |  |  |  |  |
| Vegetables | -0.003 | 0.50 | -0.038 | 0.80 |  |  |  |  |  |  |  |  |
