# Supplementary figures and images for: Gut Microbiota in Children Hospitalized with Oedematous and Non-Oedematous Severe Acute Malnutrition in Uganda
Source: PLoS Negl Trop Dis. 2016 Jan 15;10(1):e0004369. doi: 10.1371/journal.pntd.0004369 (PMC4714756; doi:10.1371/journal.pntd.0004369)

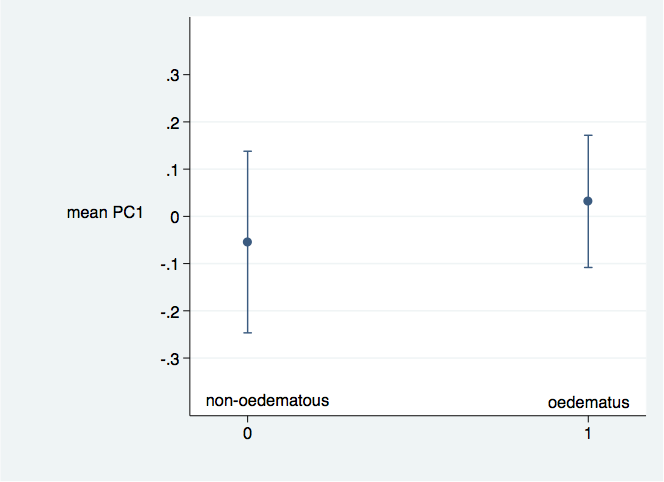

Supplement: S1 Fig — Average +/- SEM PC1 coordinate is plotted against oedematous and non-oedematous SAM children. Difference on PC1 was significant, *p = 0.0317, student’s t-test. (TIFF) [file pntd.0004369.s005.tiff]
